# Crystal structure of di­bromido­bis­(1,3-dibenzyl-1,3-diazinan-2-one-κ*O*)cobalt(II)

**DOI:** 10.1107/S2056989015014577

**Published:** 2015-08-12

**Authors:** Eduard Rais, Ulrich Flörke, René Wilhelm

**Affiliations:** a Department Chemie, Universität Paderborn, Warburgerstrasse 100, 33098 Paderborn, Germany

**Keywords:** crystal structure, coordination compound, cobalt pyrimidinone complex, C—H⋯Br inter­actions

## Abstract

The unit cell of the title complex, [CoBr_2_(C_18_H_20_N_2_O)_2_], contains 1.5 formula units per asymmetric unit with one mol­ecule sitting on a general site and a second one halved by a crystallographic twofold rotation axis passing through the Co^II^ cation. Both Co^II^ atoms are coordinated in a distorted tetra­hedral manner by two Br^−^ ligands and two O atoms of the pyrimidinone (OPyr) groups. The Br—Co—Br coordination angles are similar [115.46 (4) and 115.20 (5)°], while the O—Co—O angles differ slightly more [102.26 (18) and 98.1 (2)°]. Similarly, the Co—Br bond lengths are almost identical [2.3721 (9), 2.3757 (10) and 2.3809 (10) Å], with a larger difference between the Co—O bond lengths [1.929 (4), 1.926 (4) and 1.955 (4) Å]. The three independent OPyr groups present envelope conformations, with three C and two N atoms lying in well defined planes with maximum deviations from the least-squares planes of 0.047, 0.031 and 0.036 Å, and the external-most C atoms protruding by 0.654 (6), 0.643 (7) and 0.656 (6) Å out of the planes. The dihedral angles between the planar fractions of the OPyr planes are 50.5 (1)° for the nonsymmetric mol­ecule and 49.7 (1)° for the symmetric one. Non-covalent inter­actions are of the C—H⋯Br type and they are weak, hardly shorter than van der Waals radii, with an H⋯Br distance range of 3.00–3.04 Å. The inter­molecular inter­actions define chains parallel to [101].

## Related literature   

For cobalt complexes with urea-type ligands, see: Sone *et al.* (1984 ▸); Schafer & Curran (1966 ▸). For related *MX*_2_(OPyr)_2_ structures, see: Bobicz *et al.* (2002 ▸); Lundberg & Eriksson (2006 ▸).
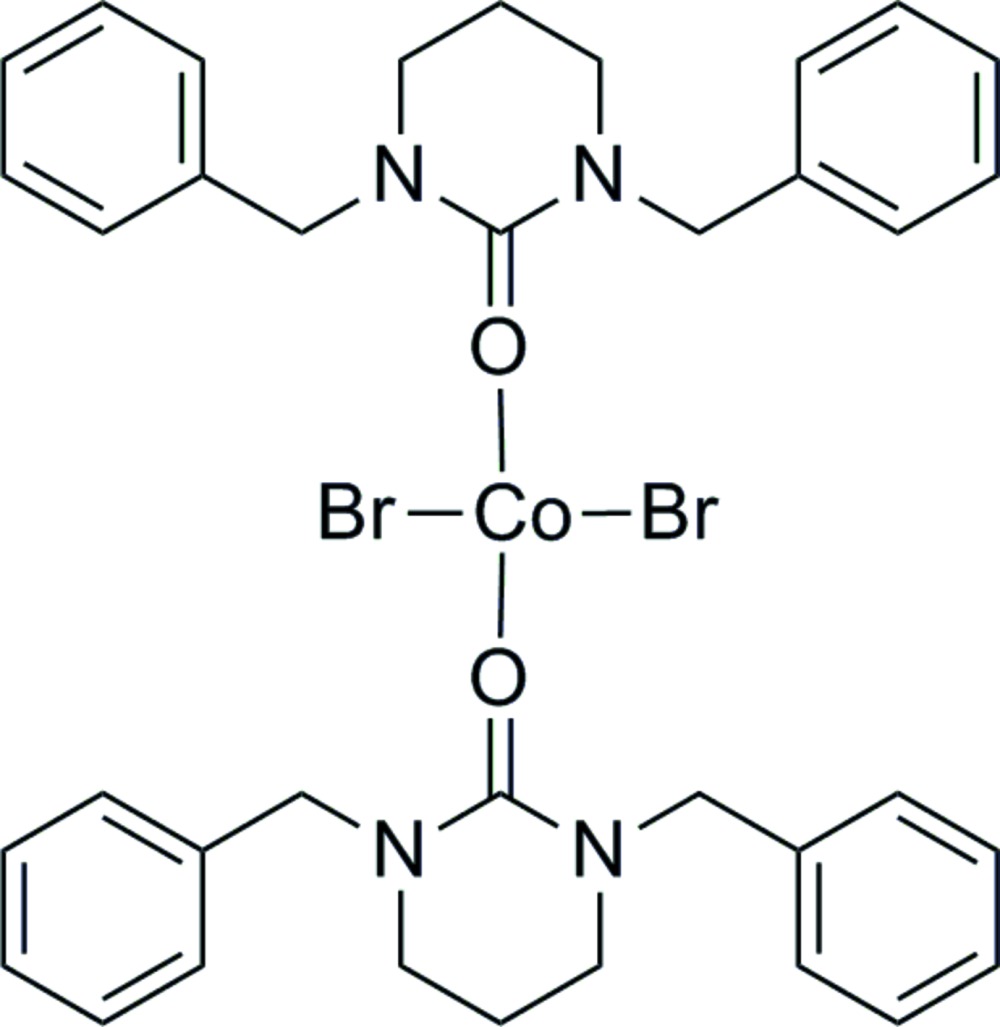


## Experimental   

### Crystal data  

[CoBr_2_(C_18_H_20_N_2_O)_2_]
*M* *_r_* = 779.47
Monoclinic, 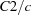

*a* = 24.182 (7) Å
*b* = 10.744 (3) Å
*c* = 40.003 (11) Å
β = 92.687 (9)°
*V* = 10382 (5) Å^3^
*Z* = 12
Mo *K*α radiation
μ = 2.84 mm^−1^
*T* = 130 K
0.47 × 0.16 × 0.11 mm

### Data collection  

Bruker SMART APEX diffractometer
Absorption correction: multi-scan (*SADABS*; Sheldrick, 2004 ▸) *T*_min_ = 0.448, *T*_max_ = 0.907
47347 measured reflections
12393 independent reflections
4968 reflections with *I* > 2σ(*I*)
*R*_int_ = 0.143

### Refinement  

*R*[*F* ^2^ > 2σ(*F* ^2^)] = 0.053
*wR*(*F* ^2^) = 0.110
*S* = 0.74
12393 reflections
609 parameters
H-atom parameters constrained
Δρ_max_ = 0.74 e Å^−3^
Δρ_min_ = −0.93 e Å^−3^

### 

Data collection: *SMART* (Bruker, 2002 ▸); cell refinement: *SAINT* (Bruker, 2002 ▸); data reduction: *SAINT*; program(s) used to solve structure: *SHELXTL* (Sheldrick, 2008 ▸); program(s) used to refine structure: *SHELXL2013* (Sheldrick, 2015 ▸); molecular graphics: *SHELXTL*; software used to prepare material for publication: *SHELXTL* and local programs.

## Supplementary Material

Crystal structure: contains datablock(s) I, global. DOI: 10.1107/S2056989015014577/bg2562sup1.cif

Structure factors: contains datablock(s) I. DOI: 10.1107/S2056989015014577/bg2562Isup2.hkl

**Fig. 1.**
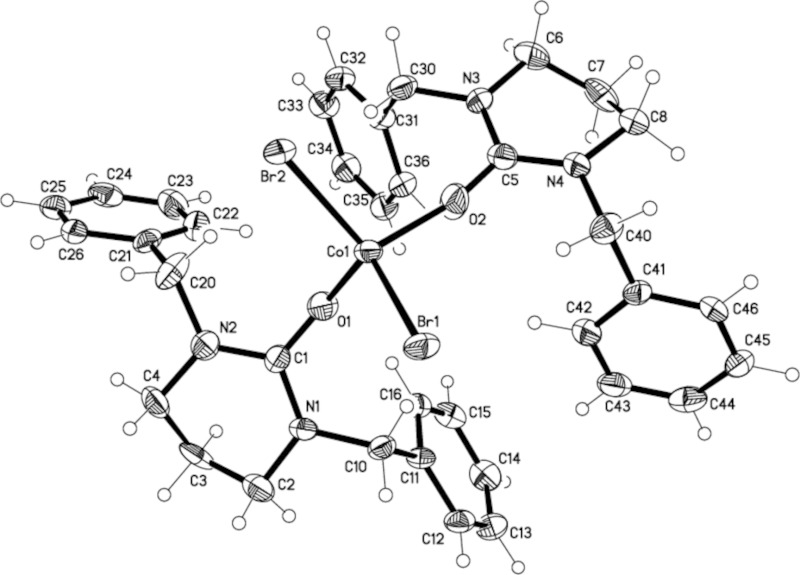
Molecular structure of molecule 1 of the title compound with anisotropic displacement ellipsoids drawn at the 50% probability level.

. DOI: 10.1107/S2056989015014577/bg2562fig1.tif
Mol­ecular structure of mol­ecule 1 of the title compound with anisotropic displacement ellipsoids drawn at the 50% probability level.

**Fig. 2.**
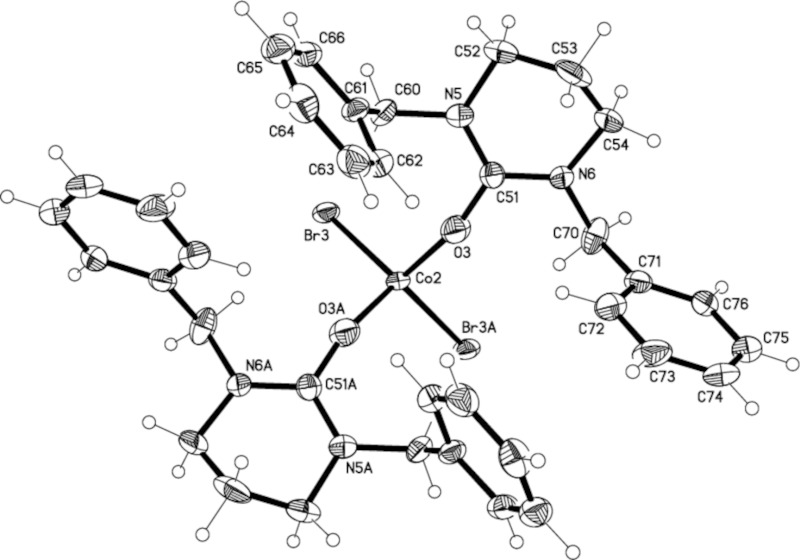
Molecular structure of molecule 2 of the title compound with anisotropic displacement ellipsoids drawn at the 50% probability level.

**Fig. 3.**
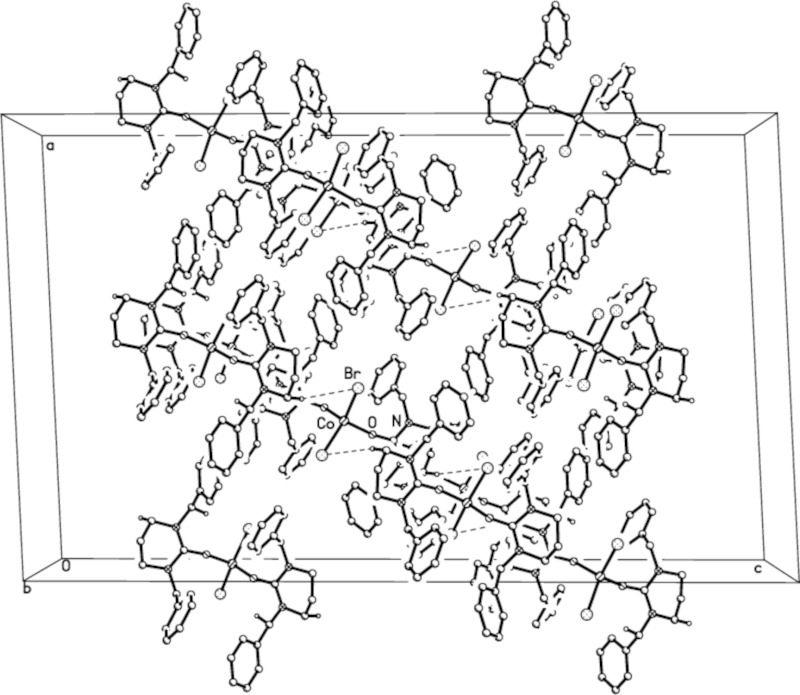
Crystal packing approximately viewed along b axis with intermolecular hydrogen bonds as dotted lines. H-atoms not involved are omitted.

. DOI: 10.1107/S2056989015014577/bg2562fig2.tif
Mol­ecular structure of mol­ecule 2 of the title compound with anisotropic displacement ellipsoids drawn at the 50% probability level.

b . DOI: 10.1107/S2056989015014577/bg2562fig3.tif
Crystal packing approximately viewed along *b* axis with inter­molecular hydrogen bonds as dotted lines. H-atoms not involved are omitted.

CCDC reference: 1416578

Additional supporting information:  crystallographic information; 3D view; checkCIF report

## Figures and Tables

**Table:** 

Co1O2	1.926(4)
Co1O1	1.955(4)
Co1Br2	2.3757(10)
Co1Br1	2.3809(10)
Co2O3	1.929(4)
Co2Br3	2.3721(9)

**Table:** 

O2Co1O1	102.26(18)
O2Co1Br2	109.10(12)
O1Co1Br2	111.60(11)
O2Co1Br1	107.58(12)
O1Co1Br1	109.93(11)
Br2Co1Br1	115.46(4)
O3^i^Co2O3	98.1(2)
O3Co2Br3	112.60(11)
O3Co2Br3^i^	108.54(11)
Br3Co2Br3^i^	115.20(5)

Symmetry code: (i) 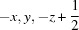.

**Table 2:** Hydrogen-bond geometry (, )

*D*H*A*	*D*H	H*A*	*D* *A*	*D*H*A*
C8H8*B*Br1^ii^	0.99	3.00	3.943(6)	159
C70H70*B*Br3^i^	0.99	3.00	3.987(6)	175
C54H54*A*Br2^iii^	0.99	3.04	3.860(6)	141

Symmetry codes: (i) 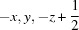; (ii) 
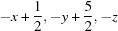
; (iii) 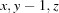.

## supplementary crystallographic information

### S1. Synthesis and crystallization

Cobalt powder (0.222 g, 3.772 mmol, 20 eq, grain size < 150 µm) and 1,3-di­benzyl-2-bromo-3,4,5,6-tetra­hydro­pyrimidin-1-ium bromide (0.080 g, 0.187 mmol, 1 eq) were filled into a schlenk tube equipped with a stirring bar. Di­methyl­formamide (3 mL) was added and the mixture was stirred and heated at 120 °C for 16 h under inert gas. The cooled down mixture was filtrated and the solvent was removed under vacuum. The residue was mixed with toluene until there was a light blue solution which was filtrated and overlayed carefully with pentane. Blue needle-shaped crystals suitable for X-ray diffraction were obtained after three days.

HR-ESI-MS [C_36_H_40_BrCoN_4_O_2_]^+^ [m/z]; calc.: 698.1667; found: 698.1673. IR(KBr) ν [cm^-1^] = 3437, 2924, 2387, 2285, 1595, 1548, 1454, 1436, 700.

### S2. Refinement

Crystalline material was of very poor quality, so various attempts were made to collect a reasonably sufficient data set. The final one still suffered from problems to give high R_int_, low ratio observed/unique reflections and a ratio data/parameters of only 8, but led to suitable refinement results.

Hydrogen atom positions were derived from geometrical considerations and then refined at idealized positions riding on the carbon atoms with isotropic displacement parameters Uiso(H) = 1.2U(Ceq) and C–H 0.95-0.99 Å.

### S3. Structural commentary

Although the present data set is only of mean quality (see Refinement Section), this very rare example of a *MX*_2_(OPyr)_2_ structure (*M* = transition metal; *X* = halogen; OPyr = pyrimidinone ligand) can be characterized adequately. Both Co centres are coordinated in a distorted tetra­hedral manner from each two Br ligands and two oxygen atoms of the pyrimidinone (OPyr) groups. The enclosed angles are Br–Co1–Br 115.46 (4)°, O–Co1–O 102.26 (19)°, Br–Co2–Br 115.20 (5)° and O–Co2–O 98.1 (2)°, as well. Co–Br bond lengths range from 2.3722 (9) to 2.3809 (10) Å, Co–O bond lengths from 1.926 (4) to 1.955 (4) Å, resp. The O–C bonds are equal with 1.253 (6) to 1.284 (6) Å and the Co–O–C angles differ strongly with 141.1 (4)° at O1 and 166.8 (4)° at O2 of molecule 1 and 152.7 (4)° at O3 of molecule 2. To date, only two other *MX*_2_(OPyr)_2_ structures are know: the *N*,*N*'-di­methyl nickel analogue (Bobicz *et al.*, 2002) and the *N*,*N*'-di­methyl di­iodo cadmium compound (Lundberg & Eriksson, 2006). Both show similar *X*–M–*X* angles of about 126°, but very different O–M–O angles of 105.83 (12)° (Ni) and 94.78 (16)° (Cd). Additionally, these structures show almost perpendicular orientation of their respectice OPyr planes, but for the two title compounds the resulting dihedral angles are 50.5 (1)° for molecule 1 and 49.7 (1)° for molecule 2. C3, C7 and C53 atoms have not been considered.

The shortest intra­molecular hydrogen bond inter­action is C20—H20A···Br2 with H···Br 2.966 Å. Inter­molecular inter­actions tightly shorter than van der Waals radii are C8–H8B···Br1(-*x* + 0.5, -*y* + 2.5, -*z*), C70–H70B···Br3(-*x*, *y*, -*z* + 0.5) and C54–H54A···Br2(*x*, *y* - 1, *z*), see Table. These link molecules into endless rows approximately elongated along [101], Figure 3.

### Crystal data

**Table:**

[CoBr_2_(C_18_H_20_N_2_O)_2_]	*F*(000) = 4764
*M**_r_* = 779.47	*D*_x_ = 1.496 Mg m^−^^3^
Monoclinic, *C*2/*c*	Mo *K*α radiation, λ = 0.71073 Å
*a* = 24.182 (7) Å	Cell parameters from 2887 reflections
*b* = 10.744 (3) Å	θ = 3.0–20.9°
*c* = 40.003 (11) Å	µ = 2.84 mm^−^^1^
β = 92.687 (9)°	*T* = 130 K
*V* = 10382 (5) Å^3^	Prism, blue
*Z* = 12	0.47 × 0.16 × 0.11 mm

### Data collection

**Table:**

Bruker SMART APEX diffractometer	12393 independent reflections
Radiation source: sealed tube	4968 reflections with *I* > 2σ(*I*)
Graphite monochromator	*R*_int_ = 0.143
φ and ω scans	θ_max_ = 27.9°, θ_min_ = 1.7°
Absorption correction: multi-scan (*SADABS*; Sheldrick, 2004)	*h* = −31→31
*T*_min_ = 0.448, *T*_max_ = 0.907	*k* = −14→13
47347 measured reflections	*l* = −52→52

### Refinement

**Table:**

Refinement on *F*^2^	Primary atom site location: structure-invariant direct methods
Least-squares matrix: full	Secondary atom site location: difference Fourier map
*R*[*F*^2^ > 2σ(*F*^2^)] = 0.053	Hydrogen site location: difference Fourier map
*wR*(*F*^2^) = 0.110	H-atom parameters constrained
*S* = 0.74	*w* = 1/[σ^2^(*F*_o_^2^) + (0.0282*P*)^2^] where *P* = (*F*_o_^2^ + 2*F*_c_^2^)/3
12393 reflections	(Δ/σ)_max_ = 0.001
609 parameters	Δρ_max_ = 0.74 e Å^−^^3^
0 restraints	Δρ_min_ = −0.93 e Å^−^^3^

### Special details

**Table:**

Geometry. All e.s.d.'s (except the e.s.d. in the dihedral angle between two l.s. planes) are estimated using the full covariance matrix. The cell e.s.d.'s are taken into account individually in the estimation of e.s.d.'s in distances, angles and torsion angles; correlations between e.s.d.'s in cell parameters are only used when they are defined by crystal symmetry. An approximate (isotropic) treatment of cell e.s.d.'s is used for estimating e.s.d.'s involving l.s. planes.

### Fractional atomic coordinates and isotropic or equivalent isotropic displacement parameters (Å^2^)

**Table:**

	*x*	*y*	*z*	*U*_iso_*/*U*_eq_	
Co1	0.16145 (3)	1.20093 (7)	0.07902 (2)	0.02788 (19)	
Br1	0.23444 (2)	1.30819 (5)	0.10883 (2)	0.03701 (16)	
Br2	0.08817 (2)	1.32827 (5)	0.05696 (2)	0.03692 (16)	
O1	0.13393 (14)	1.0661 (3)	0.10652 (10)	0.0376 (10)	
O2	0.19345 (15)	1.1117 (4)	0.04292 (10)	0.0537 (12)	
N1	0.15492 (18)	1.0075 (4)	0.16022 (11)	0.0347 (12)	
N2	0.06457 (19)	1.0539 (4)	0.14287 (12)	0.0380 (12)	
N3	0.16914 (18)	1.0201 (4)	−0.00641 (11)	0.0369 (12)	
N4	0.26004 (18)	1.0732 (4)	0.00709 (11)	0.0334 (12)	
C1	0.1179 (2)	1.0447 (5)	0.13583 (15)	0.0309 (14)	
C2	0.1386 (2)	0.9630 (5)	0.19308 (14)	0.0425 (16)	
H2A	0.1439	1.0304	0.2098	0.051*	
H2B	0.1624	0.8921	0.2003	0.051*	
C3	0.0799 (2)	0.9234 (6)	0.19150 (14)	0.0467 (17)	
H3A	0.0677	0.9070	0.2144	0.056*	
H3B	0.0757	0.8457	0.1783	0.056*	
C4	0.0448 (2)	1.0237 (6)	0.17544 (14)	0.0488 (17)	
H4A	0.0059	0.9953	0.1731	0.059*	
H4B	0.0461	1.0989	0.1898	0.059*	
C5	0.2076 (2)	1.0702 (5)	0.01531 (14)	0.0337 (14)	
C6	0.1846 (3)	0.9579 (6)	−0.03707 (14)	0.0571 (19)	
H6A	0.1809	1.0169	−0.0560	0.069*	
H6B	0.1592	0.8872	−0.0419	0.069*	
C7	0.2421 (3)	0.9118 (7)	−0.03374 (16)	0.064 (2)	
H7A	0.2445	0.8432	−0.0172	0.077*	
H7B	0.2533	0.8788	−0.0555	0.077*	
C8	0.2801 (2)	1.0131 (6)	−0.02279 (14)	0.0522 (18)	
H8A	0.3176	0.9788	−0.0178	0.063*	
H8B	0.2826	1.0751	−0.0410	0.063*	
C10	0.2137 (2)	0.9993 (5)	0.15410 (13)	0.0321 (14)	
H10A	0.2350	1.0424	0.1724	0.038*	
H10B	0.2208	1.0429	0.1329	0.038*	
C11	0.2335 (2)	0.8685 (5)	0.15192 (13)	0.0290 (13)	
C12	0.2787 (2)	0.8255 (5)	0.17192 (13)	0.0360 (15)	
H12A	0.2973	0.8803	0.1873	0.043*	
C13	0.2961 (2)	0.7043 (6)	0.16929 (15)	0.0448 (16)	
H13A	0.3275	0.6768	0.1824	0.054*	
C14	0.2690 (2)	0.6231 (5)	0.14821 (15)	0.0449 (16)	
H14A	0.2807	0.5388	0.1474	0.054*	
C15	0.2245 (2)	0.6628 (5)	0.12787 (14)	0.0422 (16)	
H15A	0.2063	0.6067	0.1126	0.051*	
C16	0.2069 (2)	0.7846 (5)	0.12998 (13)	0.0348 (14)	
H16A	0.1762	0.8118	0.1162	0.042*	
C20	0.0230 (2)	1.1003 (5)	0.11813 (16)	0.0505 (18)	
H20A	0.0415	1.1267	0.0977	0.061*	
H20B	0.0042	1.1738	0.1273	0.061*	
C21	−0.0201 (2)	0.9995 (6)	0.10897 (14)	0.0357 (15)	
C22	−0.0051 (2)	0.8939 (6)	0.09253 (15)	0.0432 (16)	
H22A	0.0317	0.8838	0.0858	0.052*	
C23	−0.0440 (3)	0.8025 (6)	0.08585 (15)	0.0513 (17)	
H23A	−0.0335	0.7276	0.0752	0.062*	
C24	−0.0969 (3)	0.8183 (6)	0.09425 (14)	0.0461 (17)	
H24A	−0.1236	0.7555	0.0890	0.055*	
C25	−0.1123 (2)	0.9238 (6)	0.11028 (14)	0.0423 (16)	
H25A	−0.1497	0.9344	0.1161	0.051*	
C26	−0.0737 (2)	1.0156 (5)	0.11811 (13)	0.0351 (14)	
H26A	−0.0841	1.0886	0.1296	0.042*	
C30	0.1112 (2)	1.0188 (5)	0.00070 (14)	0.0366 (15)	
H30A	0.0892	1.0439	−0.0197	0.044*	
H30B	0.1046	1.0809	0.0184	0.044*	
C31	0.0917 (2)	0.8945 (5)	0.01186 (13)	0.0341 (14)	
C32	0.0416 (2)	0.8423 (5)	0.00001 (14)	0.0392 (16)	
H32A	0.0189	0.8875	−0.0157	0.047*	
C33	0.0242 (2)	0.7273 (5)	0.01045 (14)	0.0414 (16)	
H33A	−0.0099	0.6939	0.0018	0.050*	
C34	0.0563 (2)	0.6608 (5)	0.03349 (15)	0.0455 (16)	
H34A	0.0445	0.5815	0.0408	0.055*	
C35	0.1050 (2)	0.7093 (5)	0.04562 (15)	0.0428 (16)	
H35A	0.1271	0.6628	0.0614	0.051*	
C36	0.1231 (2)	0.8233 (5)	0.03577 (14)	0.0385 (15)	
H36A	0.1570	0.8552	0.0451	0.046*	
C40	0.3025 (2)	1.1271 (5)	0.03009 (14)	0.0391 (15)	
H40A	0.2844	1.1793	0.0468	0.047*	
H40B	0.3271	1.1813	0.0174	0.047*	
C41	0.3368 (2)	1.0275 (5)	0.04802 (14)	0.0305 (14)	
C42	0.3118 (2)	0.9505 (5)	0.07092 (14)	0.0413 (16)	
H42A	0.2741	0.9625	0.0759	0.050*	
C43	0.3427 (3)	0.8560 (5)	0.08636 (14)	0.0443 (16)	
H43A	0.3259	0.8022	0.1018	0.053*	
C44	0.3967 (3)	0.8397 (5)	0.07969 (15)	0.0451 (17)	
H44A	0.4173	0.7744	0.0903	0.054*	
C45	0.4220 (2)	0.9173 (6)	0.05755 (15)	0.0428 (16)	
H45A	0.4599	0.9067	0.0531	0.051*	
C46	0.3910 (2)	1.0110 (5)	0.04198 (13)	0.0345 (14)	
H46A	0.4081	1.0648	0.0267	0.041*	
Co2	0.0000	0.23548 (9)	0.2500	0.0269 (3)	
Br3	−0.07599 (2)	0.11717 (5)	0.22782 (2)	0.03648 (16)	
O3	0.02697 (15)	0.3531 (3)	0.21818 (10)	0.0448 (11)	
N5	−0.00272 (18)	0.4274 (4)	0.16733 (11)	0.0351 (12)	
N6	0.09039 (18)	0.3875 (4)	0.17940 (11)	0.0329 (11)	
C51	0.0384 (2)	0.3867 (5)	0.18848 (15)	0.0334 (14)	
C52	0.0093 (2)	0.4792 (5)	0.13441 (13)	0.0432 (16)	
H52A	−0.0188	0.5428	0.1279	0.052*	
H52B	0.0074	0.4123	0.1174	0.052*	
C53	0.0653 (3)	0.5366 (6)	0.13563 (15)	0.0517 (18)	
H53A	0.0660	0.6099	0.1506	0.062*	
H53B	0.0744	0.5649	0.1130	0.062*	
C54	0.1071 (3)	0.4437 (7)	0.14817 (14)	0.059 (2)	
H54A	0.1111	0.3779	0.1311	0.070*	
H54B	0.1434	0.4850	0.1519	0.070*	
C60	−0.0591 (2)	0.4325 (5)	0.17713 (14)	0.0381 (15)	
H60A	−0.0623	0.3837	0.1979	0.046*	
H60B	−0.0832	0.3930	0.1595	0.046*	
C61	−0.0791 (2)	0.5618 (5)	0.18288 (13)	0.0314 (14)	
C62	−0.0484 (2)	0.6419 (6)	0.20404 (14)	0.0431 (16)	
H62A	−0.0155	0.6130	0.2153	0.052*	
C63	−0.0655 (2)	0.7610 (6)	0.20863 (15)	0.0474 (17)	
H63A	−0.0442	0.8144	0.2232	0.057*	
C64	−0.1130 (2)	0.8054 (5)	0.19258 (15)	0.0442 (16)	
H64A	−0.1242	0.8892	0.1957	0.053*	
C65	−0.1443 (2)	0.7279 (6)	0.17192 (15)	0.0430 (16)	
H65A	−0.1775	0.7573	0.1610	0.052*	
C66	−0.1271 (2)	0.6078 (5)	0.16719 (13)	0.0365 (15)	
H66A	−0.1488	0.5547	0.1528	0.044*	
C70	0.1342 (2)	0.3297 (5)	0.20009 (16)	0.0470 (17)	
H70A	0.1550	0.2713	0.1862	0.056*	
H70B	0.1175	0.2808	0.2180	0.056*	
C71	0.1742 (2)	0.4233 (5)	0.21587 (13)	0.0327 (14)	
C72	0.1546 (3)	0.5266 (6)	0.23188 (14)	0.0469 (17)	
H72A	0.1158	0.5395	0.2327	0.056*	
C73	0.1910 (3)	0.6120 (6)	0.24678 (15)	0.0546 (19)	
H73A	0.1773	0.6842	0.2573	0.066*	
C74	0.2466 (3)	0.5917 (6)	0.24624 (14)	0.0495 (18)	
H74A	0.2715	0.6502	0.2565	0.059*	
C75	0.2672 (2)	0.4868 (6)	0.23089 (14)	0.0433 (16)	
H75A	0.3059	0.4722	0.2309	0.052*	
C76	0.2305 (2)	0.4038 (5)	0.21561 (13)	0.0357 (14)	
H76A	0.2442	0.3323	0.2048	0.043*	

### Atomic displacement parameters (Å^2^)

**Table:**

	*U*^11^	*U*^22^	*U*^33^	*U*^12^	*U*^13^	*U*^23^
Co1	0.0283 (4)	0.0333 (5)	0.0216 (4)	−0.0008 (4)	−0.0037 (3)	0.0007 (4)
Br1	0.0366 (4)	0.0414 (4)	0.0319 (3)	−0.0063 (3)	−0.0102 (3)	−0.0002 (3)
Br2	0.0362 (4)	0.0448 (4)	0.0290 (3)	0.0082 (3)	−0.0059 (3)	0.0052 (3)
O1	0.034 (2)	0.042 (3)	0.036 (2)	−0.0061 (18)	−0.0001 (19)	0.006 (2)
O2	0.036 (3)	0.080 (3)	0.046 (3)	0.010 (2)	0.000 (2)	−0.031 (3)
N1	0.033 (3)	0.049 (3)	0.021 (3)	−0.002 (2)	−0.002 (2)	0.002 (2)
N2	0.030 (3)	0.044 (3)	0.039 (3)	−0.005 (2)	0.000 (2)	−0.003 (2)
N3	0.025 (3)	0.057 (3)	0.029 (3)	−0.002 (2)	−0.004 (2)	−0.002 (3)
N4	0.032 (3)	0.045 (3)	0.023 (3)	−0.002 (2)	0.001 (2)	0.000 (2)
C1	0.027 (3)	0.031 (3)	0.034 (4)	0.001 (3)	0.000 (3)	−0.003 (3)
C2	0.054 (4)	0.046 (4)	0.027 (4)	0.004 (3)	0.004 (3)	−0.002 (3)
C3	0.067 (5)	0.057 (5)	0.016 (3)	−0.018 (4)	0.011 (3)	−0.006 (3)
C4	0.043 (4)	0.073 (5)	0.031 (4)	−0.004 (3)	0.011 (3)	−0.020 (4)
C5	0.040 (4)	0.033 (4)	0.028 (4)	0.005 (3)	0.000 (3)	0.001 (3)
C6	0.061 (5)	0.088 (6)	0.022 (4)	0.001 (4)	−0.002 (3)	−0.013 (4)
C7	0.078 (6)	0.090 (6)	0.026 (4)	0.009 (5)	0.017 (4)	−0.015 (4)
C8	0.040 (4)	0.088 (5)	0.028 (4)	0.004 (4)	0.002 (3)	0.010 (4)
C10	0.026 (3)	0.040 (4)	0.030 (3)	−0.004 (3)	−0.005 (3)	0.004 (3)
C11	0.036 (3)	0.027 (3)	0.023 (3)	0.001 (3)	−0.004 (3)	0.005 (3)
C12	0.040 (4)	0.041 (4)	0.027 (3)	−0.002 (3)	−0.005 (3)	0.010 (3)
C13	0.045 (4)	0.049 (4)	0.039 (4)	0.013 (3)	−0.005 (3)	0.013 (3)
C14	0.059 (4)	0.031 (4)	0.045 (4)	0.009 (3)	0.006 (3)	0.005 (3)
C15	0.056 (4)	0.042 (4)	0.029 (4)	−0.006 (3)	0.002 (3)	0.000 (3)
C16	0.038 (4)	0.039 (4)	0.028 (3)	0.003 (3)	0.000 (3)	0.002 (3)
C20	0.029 (4)	0.047 (4)	0.075 (5)	0.003 (3)	−0.008 (3)	0.013 (4)
C21	0.028 (3)	0.044 (4)	0.035 (4)	0.002 (3)	−0.004 (3)	0.017 (3)
C22	0.034 (4)	0.053 (4)	0.043 (4)	0.003 (3)	0.005 (3)	0.003 (4)
C23	0.057 (5)	0.060 (5)	0.037 (4)	0.001 (4)	0.006 (3)	−0.010 (3)
C24	0.054 (4)	0.056 (4)	0.028 (3)	−0.015 (4)	−0.008 (3)	−0.007 (3)
C25	0.037 (4)	0.060 (4)	0.030 (4)	−0.009 (3)	−0.002 (3)	0.009 (3)
C26	0.032 (4)	0.046 (4)	0.028 (3)	0.002 (3)	0.002 (3)	0.006 (3)
C30	0.032 (4)	0.043 (4)	0.034 (4)	−0.001 (3)	−0.008 (3)	0.004 (3)
C31	0.037 (4)	0.035 (4)	0.029 (3)	−0.001 (3)	−0.004 (3)	−0.010 (3)
C32	0.040 (4)	0.047 (4)	0.030 (3)	0.000 (3)	−0.006 (3)	−0.002 (3)
C33	0.043 (4)	0.044 (4)	0.037 (4)	−0.009 (3)	−0.004 (3)	−0.009 (3)
C34	0.050 (4)	0.043 (4)	0.044 (4)	0.001 (3)	0.006 (3)	0.000 (3)
C35	0.042 (4)	0.043 (4)	0.044 (4)	0.009 (3)	0.004 (3)	0.009 (3)
C36	0.032 (3)	0.048 (4)	0.034 (4)	0.004 (3)	−0.004 (3)	0.001 (3)
C40	0.036 (4)	0.036 (4)	0.045 (4)	−0.006 (3)	−0.005 (3)	0.003 (3)
C41	0.029 (3)	0.035 (4)	0.027 (3)	−0.003 (3)	−0.005 (3)	−0.005 (3)
C42	0.039 (4)	0.058 (4)	0.028 (4)	−0.004 (3)	0.002 (3)	−0.003 (3)
C43	0.052 (4)	0.053 (4)	0.028 (4)	−0.007 (3)	−0.005 (3)	0.007 (3)
C44	0.058 (5)	0.039 (4)	0.037 (4)	0.011 (3)	−0.013 (3)	0.000 (3)
C45	0.030 (4)	0.061 (5)	0.037 (4)	0.006 (3)	−0.005 (3)	−0.004 (3)
C46	0.039 (4)	0.045 (4)	0.020 (3)	0.000 (3)	0.005 (3)	0.001 (3)
Co2	0.0271 (6)	0.0337 (7)	0.0194 (6)	0.000	−0.0047 (5)	0.000
Br3	0.0356 (3)	0.0454 (4)	0.0276 (3)	−0.0082 (3)	−0.0079 (3)	−0.0022 (3)
O3	0.041 (2)	0.050 (3)	0.043 (3)	−0.0133 (19)	−0.005 (2)	0.012 (2)
N5	0.035 (3)	0.041 (3)	0.029 (3)	0.002 (2)	−0.002 (2)	0.006 (2)
N6	0.027 (3)	0.045 (3)	0.026 (3)	−0.002 (2)	−0.001 (2)	−0.005 (2)
C51	0.032 (4)	0.035 (4)	0.033 (4)	−0.004 (3)	0.002 (3)	0.002 (3)
C52	0.056 (4)	0.050 (4)	0.022 (3)	0.002 (3)	−0.002 (3)	0.001 (3)
C53	0.076 (5)	0.058 (5)	0.022 (3)	−0.025 (4)	0.008 (3)	0.005 (3)
C54	0.046 (4)	0.110 (6)	0.021 (4)	−0.017 (4)	0.004 (3)	−0.007 (4)
C60	0.025 (3)	0.049 (4)	0.039 (4)	−0.002 (3)	−0.006 (3)	0.004 (3)
C61	0.028 (3)	0.035 (4)	0.030 (3)	0.002 (3)	−0.006 (3)	0.008 (3)
C62	0.032 (4)	0.063 (5)	0.034 (4)	−0.002 (3)	−0.001 (3)	−0.005 (3)
C63	0.048 (4)	0.056 (5)	0.038 (4)	−0.008 (4)	0.007 (3)	−0.012 (4)
C64	0.050 (4)	0.032 (4)	0.052 (4)	0.005 (3)	0.015 (3)	0.001 (3)
C65	0.044 (4)	0.047 (4)	0.037 (4)	0.011 (3)	−0.003 (3)	0.000 (3)
C66	0.038 (4)	0.043 (4)	0.029 (3)	−0.008 (3)	−0.006 (3)	0.003 (3)
C70	0.029 (4)	0.037 (4)	0.074 (5)	0.007 (3)	0.000 (3)	0.002 (3)
C71	0.036 (4)	0.038 (4)	0.024 (3)	0.003 (3)	−0.001 (3)	−0.002 (3)
C72	0.041 (4)	0.062 (5)	0.037 (4)	0.004 (3)	−0.004 (3)	−0.005 (3)
C73	0.054 (5)	0.071 (5)	0.038 (4)	0.018 (4)	−0.011 (3)	−0.023 (4)
C74	0.052 (4)	0.066 (5)	0.028 (4)	−0.004 (4)	−0.012 (3)	−0.006 (4)
C75	0.039 (4)	0.057 (4)	0.034 (4)	0.001 (3)	−0.003 (3)	0.005 (3)
C76	0.037 (4)	0.042 (4)	0.029 (3)	0.001 (3)	0.004 (3)	0.002 (3)

### Geometric parameters (Å, º)

**Table:**

Co1—O2	1.926 (4)	C33—C34	1.377 (7)
Co1—O1	1.955 (4)	C33—H33A	0.9500
Co1—Br2	2.3757 (10)	C34—C35	1.357 (7)
Co1—Br1	2.3809 (10)	C34—H34A	0.9500
O1—C1	1.273 (6)	C35—C36	1.364 (7)
O2—C5	1.253 (6)	C35—H35A	0.9500
N1—C1	1.354 (6)	C36—H36A	0.9500
N1—C10	1.455 (6)	C40—C41	1.515 (7)
N1—C2	1.470 (6)	C40—H40A	0.9900
N2—C1	1.336 (6)	C40—H40B	0.9900
N2—C4	1.445 (7)	C41—C46	1.355 (7)
N2—C20	1.464 (6)	C41—C42	1.393 (7)
N3—C5	1.354 (6)	C42—C43	1.388 (7)
N3—C30	1.442 (6)	C42—H42A	0.9500
N3—C6	1.460 (7)	C43—C44	1.357 (8)
N4—C5	1.325 (6)	C43—H43A	0.9500
N4—C8	1.462 (7)	C44—C45	1.379 (8)
N4—C40	1.465 (6)	C44—H44A	0.9500
C2—C3	1.480 (7)	C45—C46	1.384 (7)
C2—H2A	0.9900	C45—H45A	0.9500
C2—H2B	0.9900	C46—H46A	0.9500
C3—C4	1.497 (8)	Co2—O3^i^	1.929 (4)
C3—H3A	0.9900	Co2—O3	1.929 (4)
C3—H3B	0.9900	Co2—Br3	2.3721 (9)
C4—H4A	0.9900	Co2—Br3^i^	2.3722 (9)
C4—H4B	0.9900	O3—C51	1.284 (6)
C6—C7	1.478 (8)	N5—C51	1.347 (6)
C6—H6A	0.9900	N5—C60	1.436 (6)
C6—H6B	0.9900	N5—C52	1.471 (6)
C7—C8	1.477 (8)	N6—C51	1.326 (6)
C7—H7A	0.9900	N6—C70	1.454 (6)
C7—H7B	0.9900	N6—C54	1.461 (7)
C8—H8A	0.9900	C52—C53	1.488 (7)
C8—H8B	0.9900	C52—H52A	0.9900
C10—C11	1.489 (7)	C52—H52B	0.9900
C10—H10A	0.9900	C53—C54	1.490 (8)
C10—H10B	0.9900	C53—H53A	0.9900
C11—C16	1.394 (7)	C53—H53B	0.9900
C11—C12	1.402 (6)	C54—H54A	0.9900
C12—C13	1.375 (7)	C54—H54B	0.9900
C12—H12A	0.9500	C60—C61	1.492 (7)
C13—C14	1.360 (7)	C60—H60A	0.9900
C13—H13A	0.9500	C60—H60B	0.9900
C14—C15	1.386 (7)	C61—C66	1.385 (6)
C14—H14A	0.9500	C61—C62	1.396 (7)
C15—C16	1.381 (7)	C62—C63	1.360 (7)
C15—H15A	0.9500	C62—H62A	0.9500
C16—H16A	0.9500	C63—C64	1.375 (7)
C20—C21	1.535 (7)	C63—H63A	0.9500
C20—H20A	0.9900	C64—C65	1.375 (7)
C20—H20B	0.9900	C64—H64A	0.9500
C21—C22	1.368 (7)	C65—C66	1.371 (7)
C21—C26	1.376 (7)	C65—H65A	0.9500
C22—C23	1.378 (8)	C66—H66A	0.9500
C22—H22A	0.9500	C70—C71	1.512 (7)
C23—C24	1.349 (8)	C70—H70A	0.9900
C23—H23A	0.9500	C70—H70B	0.9900
C24—C25	1.363 (7)	C71—C72	1.377 (7)
C24—H24A	0.9500	C71—C76	1.378 (7)
C25—C26	1.384 (7)	C72—C73	1.387 (8)
C25—H25A	0.9500	C72—H72A	0.9500
C26—H26A	0.9500	C73—C74	1.362 (7)
C30—C31	1.492 (7)	C73—H73A	0.9500
C30—H30A	0.9900	C74—C75	1.388 (7)
C30—H30B	0.9900	C74—H74A	0.9500
C31—C32	1.398 (7)	C75—C76	1.380 (7)
C31—C36	1.417 (7)	C75—H75A	0.9500
C32—C33	1.377 (7)	C76—H76A	0.9500
C32—H32A	0.9500		
			
O2—Co1—O1	102.26 (18)	C33—C32—H32A	119.0
O2—Co1—Br2	109.10 (12)	C31—C32—H32A	119.0
O1—Co1—Br2	111.60 (11)	C34—C33—C32	119.8 (6)
O2—Co1—Br1	107.58 (12)	C34—C33—H33A	120.1
O1—Co1—Br1	109.93 (11)	C32—C33—H33A	120.1
Br2—Co1—Br1	115.46 (4)	C35—C34—C33	119.6 (6)
C1—O1—Co1	141.1 (4)	C35—C34—H34A	120.2
C5—O2—Co1	166.8 (4)	C33—C34—H34A	120.2
C1—N1—C10	121.0 (5)	C34—C35—C36	121.7 (6)
C1—N1—C2	122.9 (5)	C34—C35—H35A	119.1
C10—N1—C2	115.9 (4)	C36—C35—H35A	119.1
C1—N2—C4	122.4 (5)	C35—C36—C31	120.8 (5)
C1—N2—C20	121.4 (5)	C35—C36—H36A	119.6
C4—N2—C20	116.1 (5)	C31—C36—H36A	119.6
C5—N3—C30	121.4 (5)	N4—C40—C41	111.7 (4)
C5—N3—C6	121.7 (5)	N4—C40—H40A	109.3
C30—N3—C6	116.7 (5)	C41—C40—H40A	109.3
C5—N4—C8	123.4 (5)	N4—C40—H40B	109.3
C5—N4—C40	120.3 (5)	C41—C40—H40B	109.3
C8—N4—C40	116.0 (5)	H40A—C40—H40B	107.9
O1—C1—N2	121.3 (5)	C46—C41—C42	119.5 (5)
O1—C1—N1	119.8 (5)	C46—C41—C40	121.4 (5)
N2—C1—N1	118.8 (5)	C42—C41—C40	119.1 (5)
N1—C2—C3	110.7 (5)	C43—C42—C41	119.0 (6)
N1—C2—H2A	109.5	C43—C42—H42A	120.5
C3—C2—H2A	109.5	C41—C42—H42A	120.5
N1—C2—H2B	109.5	C44—C43—C42	120.7 (6)
C3—C2—H2B	109.5	C44—C43—H43A	119.7
H2A—C2—H2B	108.1	C42—C43—H43A	119.7
C2—C3—C4	109.5 (5)	C43—C44—C45	120.5 (6)
C2—C3—H3A	109.8	C43—C44—H44A	119.8
C4—C3—H3A	109.8	C45—C44—H44A	119.8
C2—C3—H3B	109.8	C44—C45—C46	118.8 (6)
C4—C3—H3B	109.8	C44—C45—H45A	120.6
H3A—C3—H3B	108.2	C46—C45—H45A	120.6
N2—C4—C3	110.1 (5)	C41—C46—C45	121.4 (6)
N2—C4—H4A	109.6	C41—C46—H46A	119.3
C3—C4—H4A	109.6	C45—C46—H46A	119.3
N2—C4—H4B	109.6	O3^i^—Co2—O3	98.1 (2)
C3—C4—H4B	109.6	O3^i^—Co2—Br3	108.54 (11)
H4A—C4—H4B	108.2	O3—Co2—Br3	112.60 (11)
O2—C5—N4	120.9 (5)	O3^i^—Co2—Br3^i^	112.60 (11)
O2—C5—N3	120.0 (5)	O3—Co2—Br3^i^	108.54 (11)
N4—C5—N3	119.1 (5)	Br3—Co2—Br3^i^	115.20 (5)
N3—C6—C7	110.8 (5)	C51—O3—Co2	152.7 (4)
N3—C6—H6A	109.5	C51—N5—C60	121.4 (5)
C7—C6—H6A	109.5	C51—N5—C52	121.0 (5)
N3—C6—H6B	109.5	C60—N5—C52	117.3 (4)
C7—C6—H6B	109.5	C51—N6—C70	121.2 (5)
H6A—C6—H6B	108.1	C51—N6—C54	122.7 (5)
C8—C7—C6	110.5 (6)	C70—N6—C54	116.1 (5)
C8—C7—H7A	109.5	O3—C51—N6	120.2 (5)
C6—C7—H7A	109.5	O3—C51—N5	119.2 (5)
C8—C7—H7B	109.5	N6—C51—N5	120.5 (5)
C6—C7—H7B	109.5	N5—C52—C53	110.2 (4)
H7A—C7—H7B	108.1	N5—C52—H52A	109.6
N4—C8—C7	110.0 (5)	C53—C52—H52A	109.6
N4—C8—H8A	109.7	N5—C52—H52B	109.6
C7—C8—H8A	109.7	C53—C52—H52B	109.6
N4—C8—H8B	109.7	H52A—C52—H52B	108.1
C7—C8—H8B	109.7	C52—C53—C54	109.6 (5)
H8A—C8—H8B	108.2	C52—C53—H53A	109.8
N1—C10—C11	112.8 (4)	C54—C53—H53A	109.8
N1—C10—H10A	109.0	C52—C53—H53B	109.8
C11—C10—H10A	109.0	C54—C53—H53B	109.8
N1—C10—H10B	109.0	H53A—C53—H53B	108.2
C11—C10—H10B	109.0	N6—C54—C53	110.8 (5)
H10A—C10—H10B	107.8	N6—C54—H54A	109.5
C16—C11—C12	118.1 (5)	C53—C54—H54A	109.5
C16—C11—C10	120.4 (5)	N6—C54—H54B	109.5
C12—C11—C10	121.5 (5)	C53—C54—H54B	109.5
C13—C12—C11	120.1 (5)	H54A—C54—H54B	108.1
C13—C12—H12A	119.9	N5—C60—C61	113.4 (5)
C11—C12—H12A	119.9	N5—C60—H60A	108.9
C14—C13—C12	121.0 (6)	C61—C60—H60A	108.9
C14—C13—H13A	119.5	N5—C60—H60B	108.9
C12—C13—H13A	119.5	C61—C60—H60B	108.9
C13—C14—C15	120.3 (6)	H60A—C60—H60B	107.7
C13—C14—H14A	119.8	C66—C61—C62	117.7 (5)
C15—C14—H14A	119.8	C66—C61—C60	122.3 (5)
C16—C15—C14	119.3 (5)	C62—C61—C60	120.0 (5)
C16—C15—H15A	120.4	C63—C62—C61	120.4 (6)
C14—C15—H15A	120.4	C63—C62—H62A	119.8
C15—C16—C11	121.1 (5)	C61—C62—H62A	119.8
C15—C16—H16A	119.4	C62—C63—C64	121.0 (6)
C11—C16—H16A	119.4	C62—C63—H63A	119.5
N2—C20—C21	111.0 (5)	C64—C63—H63A	119.5
N2—C20—H20A	109.4	C65—C64—C63	119.7 (6)
C21—C20—H20A	109.4	C65—C64—H64A	120.2
N2—C20—H20B	109.4	C63—C64—H64A	120.2
C21—C20—H20B	109.4	C66—C65—C64	119.5 (6)
H20A—C20—H20B	108.0	C66—C65—H65A	120.3
C22—C21—C26	120.3 (6)	C64—C65—H65A	120.3
C22—C21—C20	120.6 (5)	C65—C66—C61	121.7 (5)
C26—C21—C20	119.1 (6)	C65—C66—H66A	119.1
C21—C22—C23	119.4 (6)	C61—C66—H66A	119.1
C21—C22—H22A	120.3	N6—C70—C71	112.8 (5)
C23—C22—H22A	120.3	N6—C70—H70A	109.0
C24—C23—C22	120.6 (6)	C71—C70—H70A	109.0
C24—C23—H23A	119.7	N6—C70—H70B	109.0
C22—C23—H23A	119.7	C71—C70—H70B	109.0
C23—C24—C25	120.3 (6)	H70A—C70—H70B	107.8
C23—C24—H24A	119.8	C72—C71—C76	119.1 (5)
C25—C24—H24A	119.8	C72—C71—C70	120.3 (5)
C24—C25—C26	120.2 (6)	C76—C71—C70	120.5 (5)
C24—C25—H25A	119.9	C71—C72—C73	120.5 (6)
C26—C25—H25A	119.9	C71—C72—H72A	119.7
C21—C26—C25	119.1 (6)	C73—C72—H72A	119.7
C21—C26—H26A	120.5	C74—C73—C72	119.6 (6)
C25—C26—H26A	120.5	C74—C73—H73A	120.2
N3—C30—C31	113.0 (5)	C72—C73—H73A	120.2
N3—C30—H30A	109.0	C73—C74—C75	120.8 (6)
C31—C30—H30A	109.0	C73—C74—H74A	119.6
N3—C30—H30B	109.0	C75—C74—H74A	119.6
C31—C30—H30B	109.0	C76—C75—C74	118.9 (6)
H30A—C30—H30B	107.8	C76—C75—H75A	120.5
C32—C31—C36	116.1 (5)	C74—C75—H75A	120.5
C32—C31—C30	122.6 (5)	C71—C76—C75	120.9 (6)
C36—C31—C30	121.3 (5)	C71—C76—H76A	119.5
C33—C32—C31	122.0 (5)	C75—C76—H76A	119.5
			
Co1—O1—C1—N2	91.8 (7)	C36—C31—C32—C33	−1.2 (8)
Co1—O1—C1—N1	−90.7 (7)	C30—C31—C32—C33	179.8 (5)
C4—N2—C1—O1	176.6 (5)	C31—C32—C33—C34	0.6 (9)
C20—N2—C1—O1	−5.9 (8)	C32—C33—C34—C35	−0.1 (9)
C4—N2—C1—N1	−1.0 (8)	C33—C34—C35—C36	0.4 (9)
C20—N2—C1—N1	176.5 (5)	C34—C35—C36—C31	−1.1 (9)
C10—N1—C1—O1	4.1 (8)	C32—C31—C36—C35	1.4 (8)
C2—N1—C1—O1	−170.5 (5)	C30—C31—C36—C35	−179.6 (5)
C10—N1—C1—N2	−178.3 (5)	C5—N4—C40—C41	103.5 (6)
C2—N1—C1—N2	7.1 (8)	C8—N4—C40—C41	−70.2 (6)
C1—N1—C2—C3	19.8 (7)	N4—C40—C41—C46	112.1 (6)
C10—N1—C2—C3	−155.0 (5)	N4—C40—C41—C42	−67.2 (7)
N1—C2—C3—C4	−50.2 (6)	C46—C41—C42—C43	−1.8 (8)
C1—N2—C4—C3	−30.9 (7)	C40—C41—C42—C43	177.5 (5)
C20—N2—C4—C3	151.4 (5)	C41—C42—C43—C44	1.0 (9)
C2—C3—C4—N2	55.8 (6)	C42—C43—C44—C45	0.4 (9)
Co1—O2—C5—N4	119.6 (17)	C43—C44—C45—C46	−0.8 (9)
Co1—O2—C5—N3	−61 (2)	C42—C41—C46—C45	1.4 (8)
C8—N4—C5—O2	172.3 (5)	C40—C41—C46—C45	−177.9 (5)
C40—N4—C5—O2	−0.9 (8)	C44—C45—C46—C41	−0.1 (9)
C8—N4—C5—N3	−6.9 (8)	Co2—O3—C51—N6	−103.5 (8)
C40—N4—C5—N3	179.9 (5)	Co2—O3—C51—N5	80.1 (10)
C30—N3—C5—O2	3.2 (8)	C70—N6—C51—O3	11.5 (8)
C6—N3—C5—O2	−172.8 (5)	C54—N6—C51—O3	−169.6 (5)
C30—N3—C5—N4	−177.6 (5)	C70—N6—C51—N5	−172.1 (5)
C6—N3—C5—N4	6.4 (8)	C54—N6—C51—N5	6.8 (8)
C5—N3—C6—C7	23.9 (8)	C60—N5—C51—O3	−0.8 (8)
C30—N3—C6—C7	−152.3 (5)	C52—N5—C51—O3	173.4 (5)
N3—C6—C7—C8	−52.7 (7)	C60—N5—C51—N6	−177.2 (5)
C5—N4—C8—C7	−23.0 (8)	C52—N5—C51—N6	−3.0 (8)
C40—N4—C8—C7	150.5 (5)	C51—N5—C52—C53	−28.5 (7)
C6—C7—C8—N4	52.0 (7)	C60—N5—C52—C53	146.0 (5)
C1—N1—C10—C11	−107.8 (5)	N5—C52—C53—C54	54.6 (6)
C2—N1—C10—C11	67.1 (6)	C51—N6—C54—C53	21.5 (8)
N1—C10—C11—C16	53.2 (7)	C70—N6—C54—C53	−159.5 (5)
N1—C10—C11—C12	−126.0 (5)	C52—C53—C54—N6	−51.4 (7)
C16—C11—C12—C13	0.9 (8)	C51—N5—C60—C61	107.4 (6)
C10—C11—C12—C13	−179.9 (5)	C52—N5—C60—C61	−67.1 (6)
C11—C12—C13—C14	−2.1 (9)	N5—C60—C61—C66	126.7 (5)
C12—C13—C14—C15	2.7 (9)	N5—C60—C61—C62	−51.7 (7)
C13—C14—C15—C16	−1.9 (9)	C66—C61—C62—C63	−0.5 (9)
C14—C15—C16—C11	0.7 (9)	C60—C61—C62—C63	178.0 (5)
C12—C11—C16—C15	−0.1 (8)	C61—C62—C63—C64	−0.1 (9)
C10—C11—C16—C15	−179.4 (5)	C62—C63—C64—C65	0.9 (9)
C1—N2—C20—C21	117.0 (6)	C63—C64—C65—C66	−1.1 (9)
C4—N2—C20—C21	−65.3 (7)	C64—C65—C66—C61	0.4 (9)
N2—C20—C21—C22	−66.0 (7)	C62—C61—C66—C65	0.4 (8)
N2—C20—C21—C26	112.7 (6)	C60—C61—C66—C65	−178.1 (5)
C26—C21—C22—C23	−1.5 (9)	C51—N6—C70—C71	−111.2 (6)
C20—C21—C22—C23	177.2 (5)	C54—N6—C70—C71	69.9 (7)
C21—C22—C23—C24	2.5 (9)	N6—C70—C71—C72	47.9 (8)
C22—C23—C24—C25	−1.7 (9)	N6—C70—C71—C76	−134.7 (5)
C23—C24—C25—C26	−0.1 (9)	C76—C71—C72—C73	1.8 (9)
C22—C21—C26—C25	−0.3 (8)	C70—C71—C72—C73	179.2 (6)
C20—C21—C26—C25	−179.0 (5)	C71—C72—C73—C74	−1.6 (9)
C24—C25—C26—C21	1.1 (8)	C72—C73—C74—C75	0.1 (10)
C5—N3—C30—C31	−103.0 (6)	C73—C74—C75—C76	1.1 (9)
C6—N3—C30—C31	73.2 (6)	C72—C71—C76—C75	−0.5 (8)
N3—C30—C31—C32	−136.3 (5)	C70—C71—C76—C75	−177.9 (5)
N3—C30—C31—C36	44.8 (7)	C74—C75—C76—C71	−0.9 (9)

Symmetry code: (i) −*x*, *y*, −*z*+1/2.

### Hydrogen-bond geometry (Å, º)

**Table:**

*D*—H···*A*	*D*—H	H···*A*	*D*···*A*	*D*—H···*A*
C8—H8*B*···Br1^ii^	0.99	3.00	3.943 (6)	159
C70—H70*B*···Br3^i^	0.99	3.00	3.987 (6)	175
C54—H54*A*···Br2^iii^	0.99	3.04	3.860 (6)	141

Symmetry codes: (i) −*x*, *y*, −*z*+1/2; (ii) −*x*+1/2, −*y*+5/2, −*z*; (iii) *x*, *y*−1, *z*.

## References

[bb1] Bobicz, D., Kristiansson, O. & Persson, I. (2002). *J. Chem. Soc. Dalton Trans.* pp. 4201–4205.

[bb2] Bruker (2002). *SMART* and *SAINT*. Bruker AXS Inc., Madison, Wisconsin, USA.

[bb3] Lundberg, D. & Eriksson, L. (2006). *Acta Cryst.* E**62**, m400–m401.

[bb4] Schafer, M. Sr & Curran, C. (1966). *Inorg. Chem.* **5**, 265–268.

[bb5] Sheldrick, G. M. (2004). *SADABS*. University of Göttingen, Germany.

[bb6] Sheldrick, G. M. (2008). *Acta Cryst.* A**64**, 112–122. 10.1107/S0108767307043930 18156677

[bb7] Sheldrick, G. M. (2015). *Acta Cryst.* C**71**, 3–8.

[bb8] Sone, K., Kikuchi, M., Ogasawara, K., Kuya, M. K. & Pereira, A. D. (1984). *Bull. Chem. Soc. Jpn*, **57**, 3005–3006.

