# Supplementary figures and images for: Crystal structure of di­bromido­bis­(1,3-dibenzyl-1,3-diazinan-2-one-κO)cobalt(II)
Source: Acta Crystallogr E Crystallogr Commun. 2015 Aug 12;71(Pt 9):m160–1. doi: 10.1107/S2056989015014577 (PMC4555415; doi:10.1107/S2056989015014577)

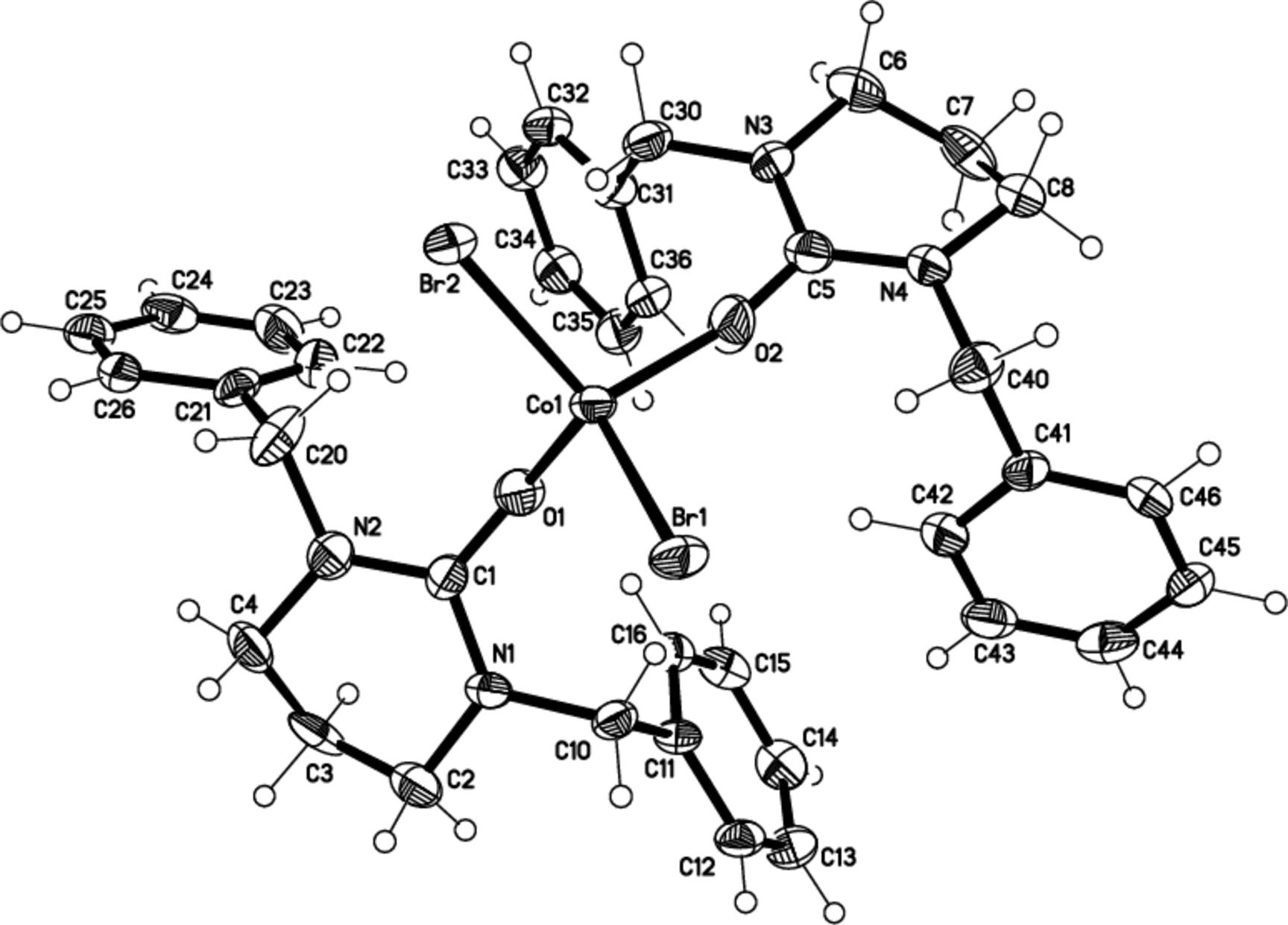

Supplement: Supplementary file 3 [file e-71-0m160-fig1.tif]

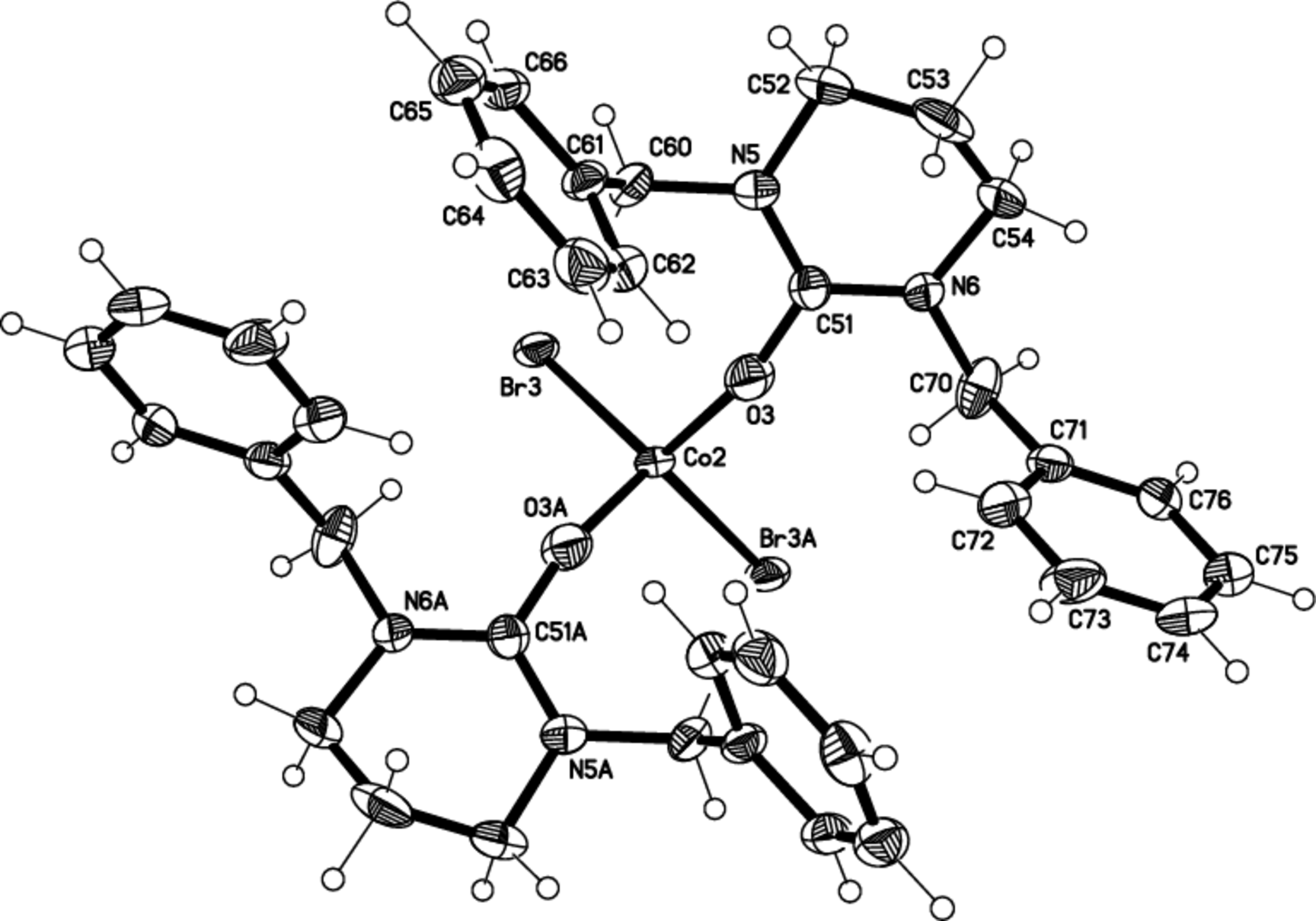

Supplement: Supplementary file 4 [file e-71-0m160-fig2.tif]

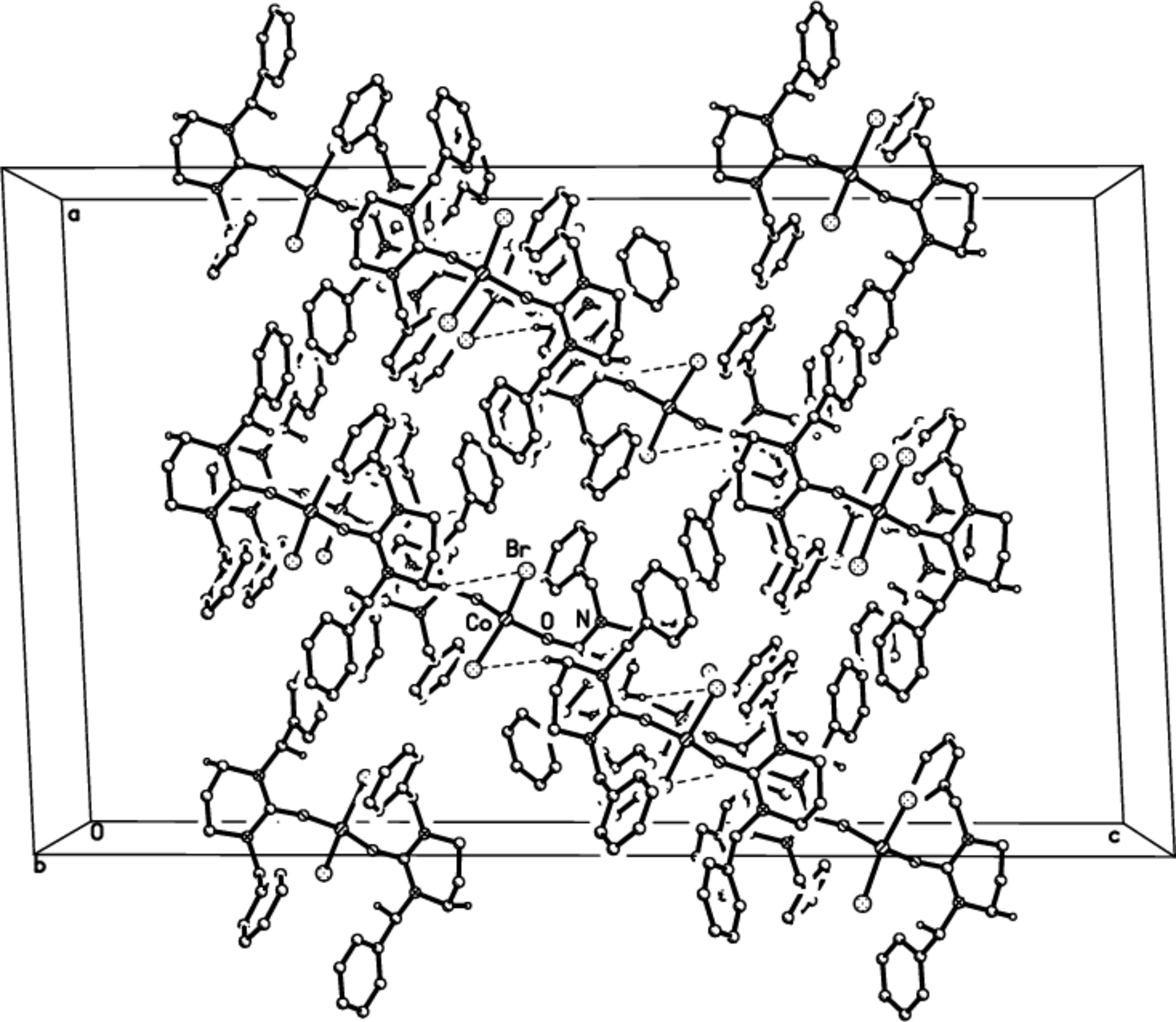

Supplement: Supplementary file 5 [file e-71-0m160-fig3.tif]
